# Delay in treatment initiation and treatment outcomes among adult patients with multidrug-resistant tuberculosis at Yangon Regional Tuberculosis Centre, Myanmar: A retrospective study

**DOI:** 10.1371/journal.pone.0209932

**Published:** 2018-12-31

**Authors:** Ye Minn Htun, Tin Mi Mi Khaing, Nay Myo Aung, Yin Yin, Zaw Myint, Si Thu Aung, Ngamphol Soonthornworasiri, Udomsak Silachamroon, Yuthichai Kasetjaroen, Jaranit Kaewkungwal

**Affiliations:** 1 Defence Services Medical School, Hmawbi, Yangon, Myanmar; 2 Regional Tuberculosis Center, Yangon, Myanmar; 3 Department of Chest Medicine, Defence Services Medical Academy, Yangon, Myanmar; 4 National Tuberculosis Programme, Ministry of Health and Sports, Nay Pyi Taw, Myanmar; 5 Department of Tropical Hygiene, Faculty of Tropical Medicine, Mahidol University, Bangkok, Thailand; 6 Department of Clinical Tropical Medicine, Faculty of Tropical Medicine, Mahidol University, Bangkok, Thailand; 7 Tuberculosis Program of the Bureau of Tuberculosis, Bangkok, Thailand; Jamia Hamdard, INDIA

## Abstract

**Background:**

Myanmar faces a health security threat, with an increasing number of multidrug-resistant tuberculosis (MDR-TB) cases. Long delays in the initiation of treatment are a barrier to MDR-TB control.

**Objectives:**

The main objectives of this study were (1) to identify the determinants of delay in treatment initiation after MDR-TB diagnosis, and (2) to explore the effects of treatment delay on disease infectivity, severity, treatment adherence, and treatment outcomes.

**Methods:**

This retrospective study reviewed 330 MDR-TB treatment cards for patients enrolled for treatment at Yangon Regional Tuberculosis Centre, in 2014.

**Results:**

Median treatment delay was 105 days, interquartile range (IQR) 106 (61–167) days; (51.5%) of patients experienced a long treatment delay (≥ 105 days). Regarding the determinants of treatment delay, this study identified important patient-healthcare system interaction factors. Significant risk factors of long treatment delay included female sex, age > 30 years, and prior contact with patients with MDR-TB. Patients with long treatment delays were significantly different from those with short delays, in terms of having high sputum smear grade, resistance to more than two main drugs (isoniazid and rifampicin), and long culture conversion time. In this study, delay in the initiation of treatment was associated with poor treatment outcome, but this was not statistically significant after adjusting for other risk factors. Median treatment-delay times were longer among patients with poor outcomes (144 days) than those with successful outcomes (102 days).

**Conclusions:**

Post-diagnosis delays in the initiation of treatment among MDR-TB patients were significantly long. The study results showed that inadequate MDR-TB treatment initiation center, centralization of treatment initiation, limitation of human resources, were health-system factors delaying timely treatment initiation and implementation of an effective TB-control program. Our findings highlight the need for immediate interventions to reduce treatment delay and improve treatment outcomes, including scaling up diagnostic capacity with Xpert MTB/RIF at township level, expansion of decentralized MDR-TB treatment initiation centers, ensuring a productive health workforce comprising trained health personnel, and providing health education and treatment-adherence counseling to patients and family members.

## Introduction

Despite the progressive control of tuberculosis (TB) worldwide, the disease burden and treatment outcomes among patients with multidrug-resistant tuberculosis (MDR-TB) have remained virtually unchanged [1]. The catastrophic socioeconomic costs incurred by patients undergoing long-term treatment is the main MDR-TB management problem in low- and middle-income countries [2]. Moreover, great challenges persist regarding the capacity of laboratories to meet the demand for scaling-up activities related to MDR-TB treatment within current TB control programs. Laboratory limitations are related to infrastructure, equipment, quality assurance, and biosafety [3]. Sputum culture and drug susceptibility testing (DST) have been endorsed for the identification of MDR-TB, but a few national TB programmes (NTPs) still have limited diagnostic capacity with respect to drug susceptibility testing (DST) for second-line drug resistance [4].

In low- and middle-income countries, expensive treatment options, limitations of second-line drugs, and many drug adverse effects, have made it increasingly difficult to treat MDR-TB. Moreover, most patients still experience long delays in accessing effective treatment, which may be months or years in countries with high MDR-TB burden [5–7]. Delays to treatment initiation have an impact at the individual level, by increasing disease progression, and at the community level, by disease transmission to others [2]. Treatment regimens for rifampicin-resistant TB (RR-TB)/MDR-TB, which last 18 months or more, are designed based on patient history or drug-resistance patterns. The World Health Organization (WHO) updated its treatment guidelines in 2016, which feature patient-centered care that is easily accessible in most settings, and social support to enable adherence through lowered costs and expected reductions in patient loss [8].

Myanmar belongs to the global list of 30 countries with a high MDR-TB burden. The expansion of services for programmatic management of drug-resistant TB (PMDT) began in 2011, based on results from the pilot Directly Observed Treatment Short course (DOTS)-PLUS project. Nonetheless, the inadequate involvement of public and private hospitals, and insufficient progress in scaling-up PMDT, have contributed barriers to the management of MDR-TB [9, 10]. The burden of MDR-TB increased significantly, with 5% estimated new cases with MDR/RR-TB and 27.1% previously treated cases, according to results of the third nationwide TB drug resistance survey in 2012–13. The WHO also estimated that approximately 9,000 cases of MDR-TB occurred in 2014 [11].

In 2013, the Myanmar NTP revised its treatment guidelines, in line with WHO recommendations, by expanding the eligibility criteria for DST and treatment. DST, liquid culture, and line probe assay (LPA) were only available at two biosafety level 3 (BSL-3) laboratories, one in Yangon (National Tuberculosis Reference Laboratory, NTRL) and the other in Mandalay (Upper Myanmar TB Laboratory, UMTBL). There was a backlog of confirmed cases who had difficulty accessing prompt treatment owing to patient limitations, limited healthcare personnel, MDR-TB diagnostic facilities, treatment centers, and the requirement of confirmation tests, although adequate second-line drugs were available for detected MDR-TB cases. A total of 68 townships expanded MDR-TB management services in 2014; however, difficulties in the scale-up of PMDT services across the nation remained a challenge. Thus, the NTP sought to scale up its diagnostic network and patient-centered care by collaborating with funding partner organizations [12, 13].

Effective MDR-TB management relies on early access to treatment; however, delays in the post-diagnosis initiation of treatment among patients is an ongoing problem [2]. It may be assumed that treatment delay contributes to poor treatment outcome. Therefore, improving the effects of delayed treatment initiation on treatment outcomes is important for interventions that enhance the linking of confirmed diagnostic results with treatment initiation. The primary objectives of this study were thus to examine the period of delay in treatment initiation after MDR-TB diagnosis, and to identify the determinants of treatment delay. The secondary objective was to explore the effects of treatment delay on disease infectivity, severity, treatment adherence, and treatment outcomes.

## Materials and methods

### Study setting

**Fig 1:**
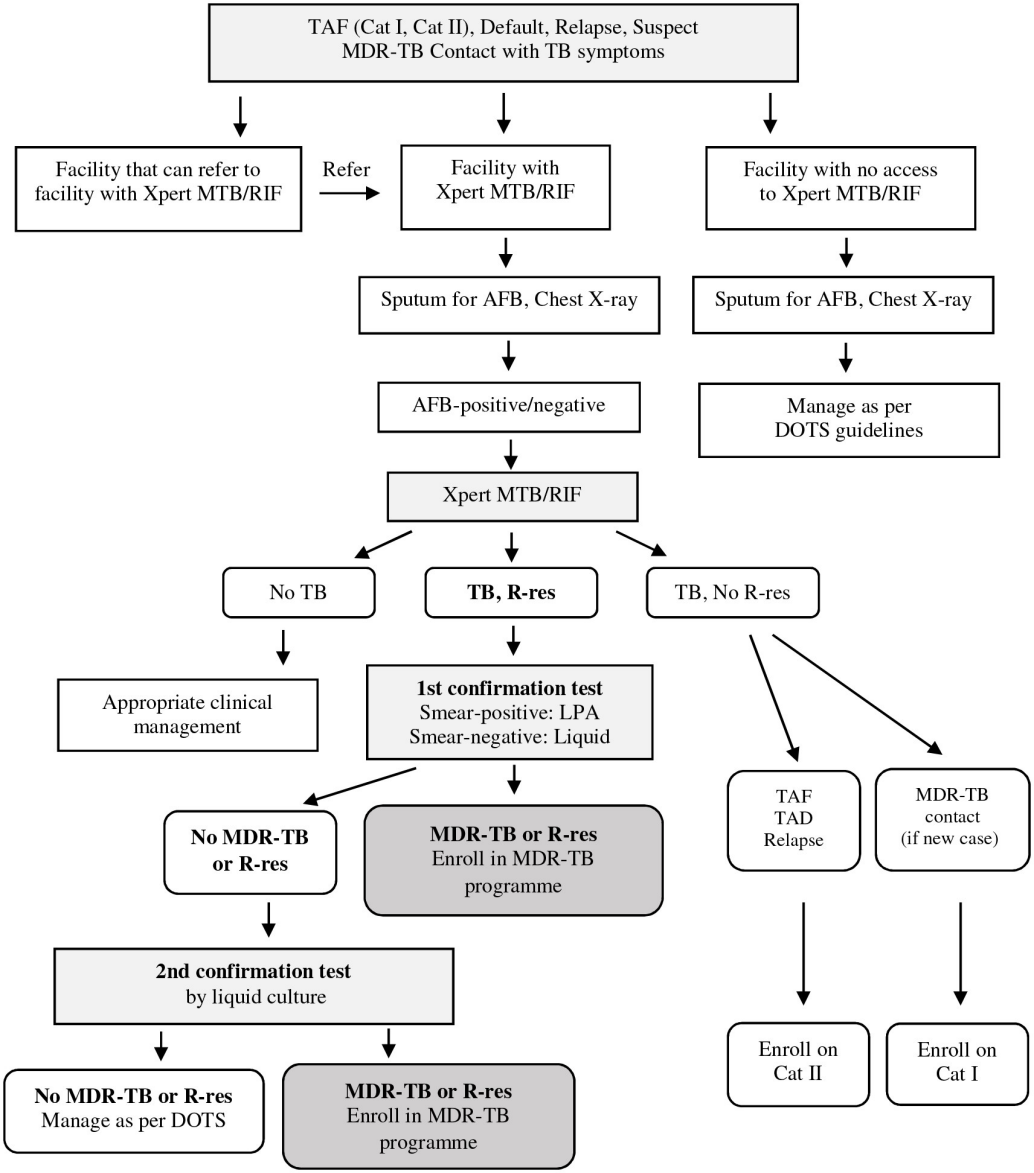
Diagnosis of MDR-TB in patients with risk factors for resistance. R-res; Rifampicin Resistance. TAF-I; Treatment after Failure Category I. TAF-II; Treatment after Failure Category II. LPA; Line probe Assay. Cat I; Treatment for new patients with first-line anti-TB drugs (2HREZ/4HR). Cat II; Re-treatment regimen with first-line drugs (2SHREZ/1HREZ/3HRE).

Yangon Region has 4 districts, including 46 townships, with a total population of approximately 7.3 million people [14]. In 2014, the main challenges of Yangon Regional TB Centre included high population density, inadequate health facilities for early treatment initiation, poor electronic recording/reporting, and insufficient funding for confirmed MDR-TB cases. During that period, treatment initiation for confirmed MDR-TB cases was centralized at the Regional TB Center. The diagnostic algorithms of the national guidelines followed the WHO recommendations [15–18]. For MDR-TB diagnosis, the Township Medical Officer (TMO)/Township TB Coordinator identified all patients with treatment failure (Category I, Category II), default, or relapse; those who had previous contact with MDR-TB patients with TB symptoms; and TB patients living with HIV/AIDS (Fig 1). After first conducting a sputum examination for acid-fast bacilli (AFB) and chest X-ray in the Township Health Centre, two sputum specimens of patients with positive or negative results by sputum AFB testing were sent to the regional TB laboratory for Xpert MTB/RIF testing, as an initial diagnostic test. The smear was stained using the Ziehl-Neelsen method, examined with a bright-field binocular microscope, and graded according to the WHO grading chart for AFB microscopy. Fluorescence microscopy is also used instead of bright-field microscopy, especially at times of high microscopy workload [15].

If RR-TB was detected, a second sputum specimen was sent to the NTRL for a first confirmatory test, using LPA, Genotype MTBDR *plus* Test (Hain Life Sciences), or liquid culture and DST. If RR-TB was not confirmed by the results of the first test, a second confirmatory test was performed with liquid culture. Liquid culture and DST was performed by Mycobacterium Growth Indicator Tube system (MGIT-960R) using liquid medium (Middlebrook 7H9 broth). DST was performed in the same MGIT machine, from an inoculum of liquid culture-positive tubes into the drug-containing tubes [15]. For HIV-negative patients with suspected TB, a different procedure was followed in the Township Health Centers. First, a sputum AFB test was performed; chest X-rays would be performed only for patients with positive sputum AFB results. If the chest X-ray findings were abnormal, sputum specimens were sent to the regional TB laboratory for Xpert MTB/RIF, following the process described above.

In the Yangon Region, there were six Xpert MTB/RIF machines for initial screening, in 2014; however, the mainstay confirmatory tests for MDR-TB were liquid culture and DST or LPA. Specimens were transported by Basic Health Staff (BHS) or patients themselves to Xpert MTB/RIF centers. The confirmed results were conveyed by both telephone and post to the Regional TB Center within 1–3 weeks, and were then sent to the township medical officer (TMO). Patients received confirmed results from the TMO via the BHS [15]. Patients with laboratory-confirmed MDR-TB were routinely enrolled in a second-line anti-TB drug treatment program. An Expert Drug Resistant-TB (DR-TB) Committee made the decision to start treatment for confirmed cases, during their monthly committee meeting. The patients selected for treatment were referred to the Regional TB Centre after counseling by BHS or the TMO about the importance of regular treatment and possible side effects.

Before starting treatment, patients were registered and baseline investigations (complete blood count, liver function, renal function, thyroid function, blood sugar, serum electrolytes, pregnancy) were conducted at the Regional TB Centre [11, 15]. Treatment adherence counseling and health education were also provided to patients and their family members by a trained counselor (health staff, such as BHS, the TB Coordinator, Medical Officers, or a medical social worker) from the Regional TB Centre or Township Health Centre. The patients were registered in the township MDR-TB register to continue treatment in their respective townships. Patients attended monthly follow-up visits at the Regional TB Centre to monitor treatment response [15].

All patients with MDR-TB were treated with a standardized regimen, based on the results of the MDR-TB pilot DOTS-PLUS project. This regimen comprised 6–8 months of the intensive phase, using injectable amikacin, pyrazinamide, levofloxacin, ethionamide, and cycloserine, followed by 12–14 months of the continuous phase using levofloxacin, ethionamide, cycloserine, and pyrazinamide. Both the duration of the intensive phase and the total duration of treatment were guided by culture conversion [11, 15]. The treatment was provided by DOTS providers or midwives as an ambulatory service with daily provision of injections, treatment monitoring, counseling, and contact tracing. Medicines were supplied free of charge to all patients [11].

Treatment response was monitored by monthly sputum smear examination, and culturewas done of the same specimen for the months that culture was indicated. A combination of solid (at months 3, 9, 12, 14, and 18) and liquid culture (at months 5, 6, 7, 8, 16, and 20 or 24) was used to monitor treatment. Treatment outcomes were based on the results of sputum smear and mycobacterial culture as monitoring tools, in line with WHO guidelines. Continued monthly clinical and laboratory follow-up was provided free of charge [15].

### Study population and design

We conducted a retrospective study in 2017. Based on the database of the Yangon Regional TB Centre, systematic random sampling technique was employed to select 330 patients from among 997 treatment cards of patients (cases) registered and enrolled for treatment in 2014. A list of patients with MDR-TB served as a sampling frame, and we used a sampling interval of three to obtain the required sample size. Inclusion criteria: patient age > 18 years with pulmonary involvement and follow-up treatment outcome; if a patient was aged < 18 years, we selected a subsequent patient. Thus, all selected patients had complete follow-up data and treatment outcomes as available in the database structure (S1 File). We extracted patient data manually, including the date of DST, date of registration at the Regional TB Centre, date of treatment initiation, demographics, disease factors (type of patient, contact with patients who had MDR-TB, diabetes mellitus, HIV status, lung cavitation, smear grade, drug resistance pattern, treatment duration, culture conversion time, and treatment outcomes), as well as information about geographic accessibility.

### Definition of variables

The definition of delay in treatment initiation was based on the WHO definition [19]. Patient categories and treatment outcomes were in line with NTP guidelines, which were also adopted from the WHO definitions [15, 20].

#### Treatment delay

In this study, we defined delay in treatment initiation as the duration in days between the date of MDR-TB confirmation and the date of treatment initiation. We categorized delay in treatment as short (< median) and long (≥ median), according to the median cutoff point.

#### Types of MDR-TB patient

We classified patients with MDR-TB into two categories: new case and previously treated case, according to the definition and reporting framework for tuberculosis (2013 revision) [20]. New cases were patients who had never been treated for TB or had taken anti-TB drugs for < 1 month. Previously treated patients were patients who had received ≥ 1 month of anti-TB drug treatment in the past, including relapsed patients, those with treatment after Category I and Category II failure, those with standard MDR-TB regimen failure, and default patients [15, 20].

#### BMI

We defined underweight patients as those with body mass index (BMI) < 18.5 kg/m^2^ (weight in kilograms divided by the square of height in meters). We categorized patients as BMI groups < 18.5 and ≥ 18.5 kg/m^2^.

#### Sputum smear grading

In this study, we dichotomized sputum AFB smear grading as low grade, which was combined negative (no AFB in at least 100 fields), 1+ (10 to 99 AFB in 100 fields), and high grade, which was combined 2+ (1 to 10 AFB per field in at least 50 fields) and 3+ (> 10 AFB per field in at least 20 fields) [21].

#### Culture conversion

We defined sputum culture conversion as the first date of two consecutive negative cultures, taken at least 30 days apart, and categorized culture conversion time as short (< median) and long (≥ median), according to the median cutoff point.

#### Treatment outcomes

We classified treatment outcomes into two categories: successful (a combination of cured and treatment completed) and poor (a combination of failed, died, lost to follow-up, and not evaluated).

#### Treatment adherence

By programmatic management of drug resistance TB (PMDT), patients were monitored for treatment adherence on a monthly basis. Patients who had complete follow-up visits referred to those who came to all appointed and required number of visits; they generally included those who were cured, complete treatment, failed or not evaluated. Patients with incomplete adherence status referred to those who were lost to follow-up or died prior to treatment completion; they were also counted as part of those with poor treatment outcomes.

### Data analysis

We entered the data into Microsoft Excel and cleaned the data before analysis. IBM SPSS version 23.0 for Windows (IBM Corp., Armonk, NY, USA) was used for analysis. We reported continuous variables as mean ± standard deviation (SD) or median with interquartile range (IQR), and categorical variables were reported as proportions, to describe patient characteristics. The delay period was expressed using Kaplan–Meier curves. We used binary regression models to assess the independent predictors of long treatment delay and poor treatment outcome. We performed univariate analysis on each independent variable (including demographic factors, disease factors, and geographical accessibility) and calculated the respective crude risk ratio (CRR). We also conducted multivariate analysis for all variables in univariate analysis and calculated the respective adjusted risk ratio (ARR).

In addition, we explored the associations between treatment delay and other determinants, including lung cavitation, sputum-smear grading, resistance pattern, and culture conversion using cross-tabulation, and expressed these as relative risk ratio (RR). The log-rank test was used to compare median treatment delay between patients who achieved successful and poor outcomes. The Chi square test was used to determine the association between treatment delay and treatment adherence, which was classified as complete or incomplete visits during the patients’ routine monthly follow-ups. For all statistical tests, we calculated 95% confidence intervals (CI) and considered a *p*-value < 0.05 to indicate statistical significance.

### Ethical considerations

The study was approved by the Ethics Committee, Office of Research Services, Faculty of Tropical Medicine, Mahidol University, Thailand (MUTM 2017-030-01) and the Institutional Review Board of the Defence Services Medical Research Centre, Myanmar (IRB/2017/65), which approved waiver of patient signed informed consent. Permission to review patient treatment cards and extract data was also obtained from the National Tuberculosis Program (NTP), Department of Public Health, Ministry of Health and Sports (MOHS), Myanmar (Letter No. 268/TB). The data were extracted and de-identified by an authorized person who was a member of the study team. The research team restricted sharing the data publicly to protect the confidentiality of patient information and the medical-data security of the NTP.

## Results

### Characteristics of patients with MDR-TB

The cohort consisted of 330 patients with MDR-TB who were enrolled for treatment in 2014. Of these, 64.8% were male and 35.2% female, i.e., a sex ratio of 1.8:1. The mean (± SD) age for all patients was 39.45 (± 13.28) years, with range 18–77 years. Nearly half of patients were aged 31–50 years. Mean (± SD) BMI was 20.96 (± 2.79) kg/m^2^, with range 10.70–26.20 kg/m^2^, and 25.8% of patients were underweight (BMI < 18.5 kg/m^2^). Most patients (74.8%) were living in urban areas and 25.2% in rural areas (Table 1).

**Table 1:** Characteristics of patients with MDR-TB.

Variables	Number (%)
**Demographic Factors**	
Sex	
Male	214 (64.8)
Female	116 (35.2)
Age	
≤ 30 years	99 (30.0)
31–50 years	162 (49.1)
> 50 years	69 (20.9)
Mean ± SD (39.45±13.28), Minimum 18, Maximum 77	
BMI	
< 18.5	85 (25.8)
≥ 18.5	245 (74.2)
Mean ± SD (20.96±2.79), Minimum 10.70, Maximum 26.20	
**Geographical Accessibility**	
Residence	
Rural	83 (25.2)
Urban	247 (74.8)
**Disease Factors**	
Type of patient	
New case	35 (10.6)
Previously treated case	295 (89.4)
Relapse	154 (52.2)
Treatment after failure (Category I)	70 (23.7)
Treatment after failure (Category II)	67 (22.7)
Default	4 (1.2)
Contact with MDR-TB patient	
Absent	212 (64.2)
Present	118 (35.8)
Diabetes mellitus	
Absent	301 (91.2)
Present	29 (8.8)
HIV coinfection	
Absent	298 (90.3)
Present	32 (9.7)
Lung cavitation	
Absent	307 (93.0)
Present	23 (7.0)
Unilateral	16 (69.6)
Bilateral	7 (30.4)
Smear grade	
Negative	28 (8.5)
1+	142 (43.0)
2+	66 (20.0)
3+	94 (28.5)
Drug-resistance pattern	
HR	296 (89.7)
SHR	18 (5.5)
SHRE	16 (4.8)
Treatment duration (n = 269)	
20 months	230 (85.5)
24 months	39 (14.5)
Culture conversion time (n = 269)	
Short (< 152 Days)	117 (43.5)
Long (≥ 152 Days)	152 (56.5)
Median 152, IQR 147 (94–241), Minimum 90, Maximum 365	
Treatment outcome (n = 330)	
Successful	269 (81.5)
Cured	210 (63.6)
Completed	59 (17.9)
Poor	61 (18.5)
Lost to follow-up	9 (0.03)
Failed	3 (0.01)
Died	48 (14.5)
Not evaluated	1 (0.00)

Abbreviations: MDR-TB, multidrug-resistant tuberculosis; SD, standard deviation; BMI, body mass index; IQR, interquartile range; S, streptomycin; H, isoniazid; R, rifampin; E, ethambutol.

Among all patients with MDR-TB, 10.6% were new cases and 89.4% were previously treated. Of the total patients, 35.8% had a history of contact with other patients who had MDR-TB; 8.8% of patients had diabetes mellitus, 9.7% had HIV coinfection, and 7% had lung cavitation. Of all patients, 43% had sputum smear grade (1+) at treatment initiation and 89.7% were resistant to HR (isoniazid and rifampicin) drugs only. Most patients (85.5%) were treated for 20 months and the remainder for 24 months. The median time to culture conversion was 152 days, IQR 147 (94–241), and 56.5% of patients had long sputum culture conversion times (≥ 152 days) after treatment initiation. 81.5% of patients achieved successful outcomes and the remainder poor outcomes.

### Treatment delay and associated factors in patients with MDR-TB

**Fig 2:**
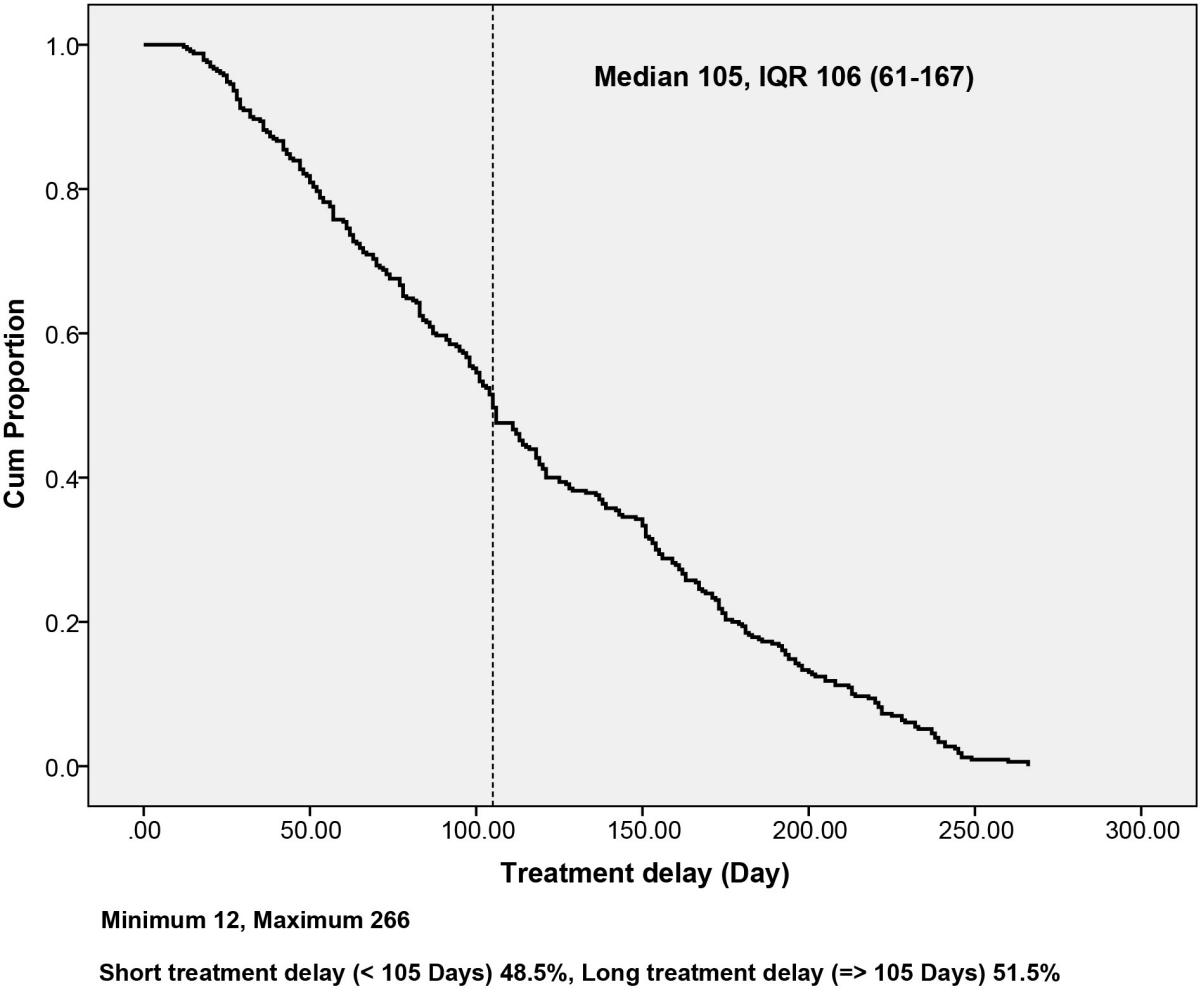
Treatment delay among patients with MDR-TB.

The median treatment delay was 105 days, with IQR 106 (61–167) days; 51.5% of patients experienced long treatment delays (Fig 2). Among all participants, female patients experienced longer treatment delays (ARR = 1.40, 95% CI = 1.03–1.91) than male patients. Compared with patients aged < 30 years, those aged 31–50 years had longer treatment delays (ARR = 1.71, 95% CI = 1.15–2.57); patients aged > 50 years also had longer treatment delays (ARR = 1.77, 95% CI = 1.11–2.82). Patients reporting a history of contact with a patient who had MDR-TB had longer treatment delays (ARR = 1.48, 95% CI = 1.06–2.08) than patients with no contact history (Table 2). In all, female sex, age > 30 years, and previous contact with a patient who had MDR-TB, were significant risk factors for long treatment delay (*p* < 0.05).

**Table 2:** Factors associated with treatment delay among patients with MDR-TB.

Variables	Treatment Delay n (%)		CRR (95% CI)	ARR (95% CI)
	Short delay	Long delay		
Sex				
Male	120 (56.1)	94 (43.9)	1.00	1.00
Female	40 (34.5)	76 (65.5)	1.49 (1.10–2.02) *	1.40 (1.03–1.91) *
Age				
≤ 30 years	66 (66.7)	33 (33.3)	1.00	1.00
31–50 years	66 (40.7)	96 (59.3)	1.78 (1.19–2.64) *	1.71 (1.15–2.57) *
> 50 years	28 (40.6)	41 (59.4)	1.78 (1.13–2.82) *	1.77 (1.11–2.82) *
Type of patient				
New case	9 (25.7)	26 (74.3)	1.00	1.00
Previously treated case	151 (51.2)	144 (48.8)	0.66 (0.43–0.99) *	0.93 (0.58–1.50)
Contact with MDR-TB patient				
Absent	123 (58.0)	89 (42.0)	1.00	1.00
Present	37 (31.4)	81 (68.6)	1.64 (1.21–2.21) *	1.48 (1.06–2.08) *
Diabetes mellitus				
Absent	152 (50.5)	149 (49.5)	1.00	1.00
Present	8 (27.6)	21 (72.4)	1.46 (0.93–2.31)	1.09 (0.68–1.76)
HIV coinfection				
Absent	150 (50.3)	148 (49.7)	1.00	1.00
Present	10 (31.3)	22 (68.8)	1.38 (0.89–2.17)	1.38 (0.87–2.18)
Residence				
Urban	129 (52.2)	118 (47.8)	1.00	1.00
Rural	31 (37.3)	52 (62.7)	1.31 (0.95–1.82)	1.25 (0.89–1.73)

Sex, age, type of patient, contact with MDR-TB patient, diabetes mellitus, HIV coinfection, and residence were included in the model. Treatment delay was categorized into short (< 105 days) and long (≥ 105 days). Abbreviations: MDR-TB, multidrug-resistant tuberculosis; CRR, crude risk ratio; ARR, adjusted risk ratio.

* *p* value < 0.05 was significant

### Effects of delay in treatment initiation on disease infectivity, severity, and treatment efficacy

Patients with a long treatment delay were 41% more likely to have a high smear grade (RR = 1.41, 95% CI = 1.12–1.78) than patients with a short treatment delay (Table 3). Patients who had long treatment delays were three times more likely to have resistance to > HR drugs (RR = 3.06, 95% CI = 1.43–6.56) and 49% more likely to have long culture conversion times (RR = 1.49, 95% CI = 1.19–1.85) than patients with short treatment delays. These associations were statistically significant (*p* < 0.05).

**Table 3:** Effects of treatment delay on disease infectivity, severity, and treatment efficacy.

Treatment Delay	Lung Cavitation ᶲ n (%)		RR (95% CI)	Smear Grade * n (%)		RR (95% CI)	Resistance Pattern * n (%)		RR (95% CI)	Culture Conversion * n (%)		RR (95% CI)
	Present	Absent		High	Low		> HR	HR		Long	Short	
Long	14	156	1.46	96	74	1.41	26	144	3.06	89	42	1.49
	(8.2)	(91.8)	(0.65–3.29)	(56.5)	(43.5)	(1.12–1.78)	(15.3)	(84.7)	(1.43–6.56)	(67.9)	(32.1)	(1.19–1.85)
Short	9	151	1.00	64	96	1.00	8	152	1.00	63	75	1.00
	(5.6)	(94.4)		(40.0)	(60.0)		(5.0)	(95.0)		(45.7)	(54.3)	
Total	23	307		160	170		34	296		152	117	
	(7.0)	(93.0)		(48.5)	(51.5)		(10.3)	(89.7)		(56.5)	(43.5)	

Resistance pattern was categorized as HR (resistance to isoniazid and rifampicin only) and > HR (resistance to SHR and SHRE). Treatment delay was categorized as short delay (< 105 days) and long delay (≥ 105 days). Abbreviations: CI, confidence interval; RR, risk ratio.

^ᶲ^ *p* value 0.39,

* *p* value < 0.05 Significant

### Effect of delay in treatment initiation on treatment outcome

As shown in Table 4, more patients with long treatment delay had poor outcomes than patients with short treatment delay (CRR = 1.69, 95% CI = 1.08–2.78); however, after adjusting for other factors, the association between delay in treatment initiation and treatment outcomes was not statistically significant. Patients with BMI ≥ 18.5 kg/m^2^ had less risk of a poor outcome (ARR = 0.33, 95% CI = 0.19–0.56) than those with BMI < 18.5 kg/m^2^ (*p* < 0.001). Patients with HIV coinfection had a nearly four times greater risk of a poor outcome (ARR = 3.85, 95% CI = 2.16–6.86) than those without HIV infection (*p* < 0.001). Patients with high smear grade (≥ 2+) had a 75% greater risk of a poor outcome (ARR = 1.75, 95% CI = 1.01–3.02) than patients with a low smear grade (negative and 1+) (*p* < 0.05).

**Table 4:** Factors associated with poor treatment outcome among patients with MDR-TB.

Variables	Treatment Outcomes		CRR (95% CI)	ARR (95% CI)
	Successful n (%)	Poor n (%)		
Treatment delay				
Short	138 (86.3)	22 (13.8)	1.00	1.00
Long	131 (77.1)	39 (22.9)	1.69 (1.08–2.78) *	1.11 (0.63–1.97)
Sex				
Male	172 (80.4)	42 (19.6)	1.00	1.00
Female	97 (83.6)	19 (16.4)	0.84 (0.49–1.44)	0.69 (0.39–1.22)
Age				
≤ 30 years	85 (85.9)	14 (14.1)	1.00	1.00
31–50 years	131 (80.9)	31 (19.1)	1.35 (0.72–2.54)	1.19 (0.61–2.34)
> 50 years	53 (76.8)	16 (23.2)	1.64 (0.80–3.36)	1.89 (0.84–4.25)
BMI				
< 18.5	49 (57.6)	36 (42.4)	1.00	1.00
≥ 18.5	220 (89.8)	25 (10.2)	0.24 (0.15–0.40) **	0.33 (0.19–0.56) **
Type of patient				
New case	24 (68.6)	11 (31.4)	1.00	1.00
Previously treated case	245 (83.1)	50 (16.9)	0.53 (0.28–0.98) *	0.59 (0.29–1.18)
Diabetes mellitus				
Absent	250 (83.1)	51 (16.9)	1.00	1.00
Present	19 (65.5)	10 (34.5)	2.03 (1.03–4.01) *	1.33 (0.62–2.81)
HIV co-infection				
Absent	259 (86.9)	39 (13.1)	1.00	1.00
Present	10 (31.3)	22 (68.8)	5.25 (3.12–8.86) **	3.85 (2.16–6.86) **
Lung cavitation				
Absent	250 (81.4)	57 (18.6)	1.00	1.00
Present	19 (82.6)	4 (17.4)	0.94 (0.34–2.58)	0.84 (0.29–2.39)
Resistance pattern				
HR	241 (81.4)	55 (18.6)	1.00	1.00
> HR	28 (82.4)	6 (17.6)	0.95 (0.41–2.21)	0.93 (0.39–2.19)
Smear grade				
Low	148 (87.1)	22 (12.9)	1.00	1.00
High	121 (75.6)	39 (24.4)	1.88 (1.12–3.18) *	1.75 (1.01–3.02) *

All patients who achieved culture conversion were included as having a successful outcome. Treatment delay, sex, age, BMI, type of patient, diabetes mellitus, HIV coinfection, lung cavitation, resistance pattern, and smear grade were included in the model. Treatment delay was categorized as short (< 105 days) and long (≥ 105 days). Abbreviations: MDR-TB, multidrug-resistant tuberculosis; CRR, crude risk ratio; ARR, adjusted risk ratio; CI, confidence interval; BMI, body mass index; H, isoniazid; R, rifampicin.

* *p* value < 0.05 and

** *p* value < 0.001 Significant

**Fig 3:**
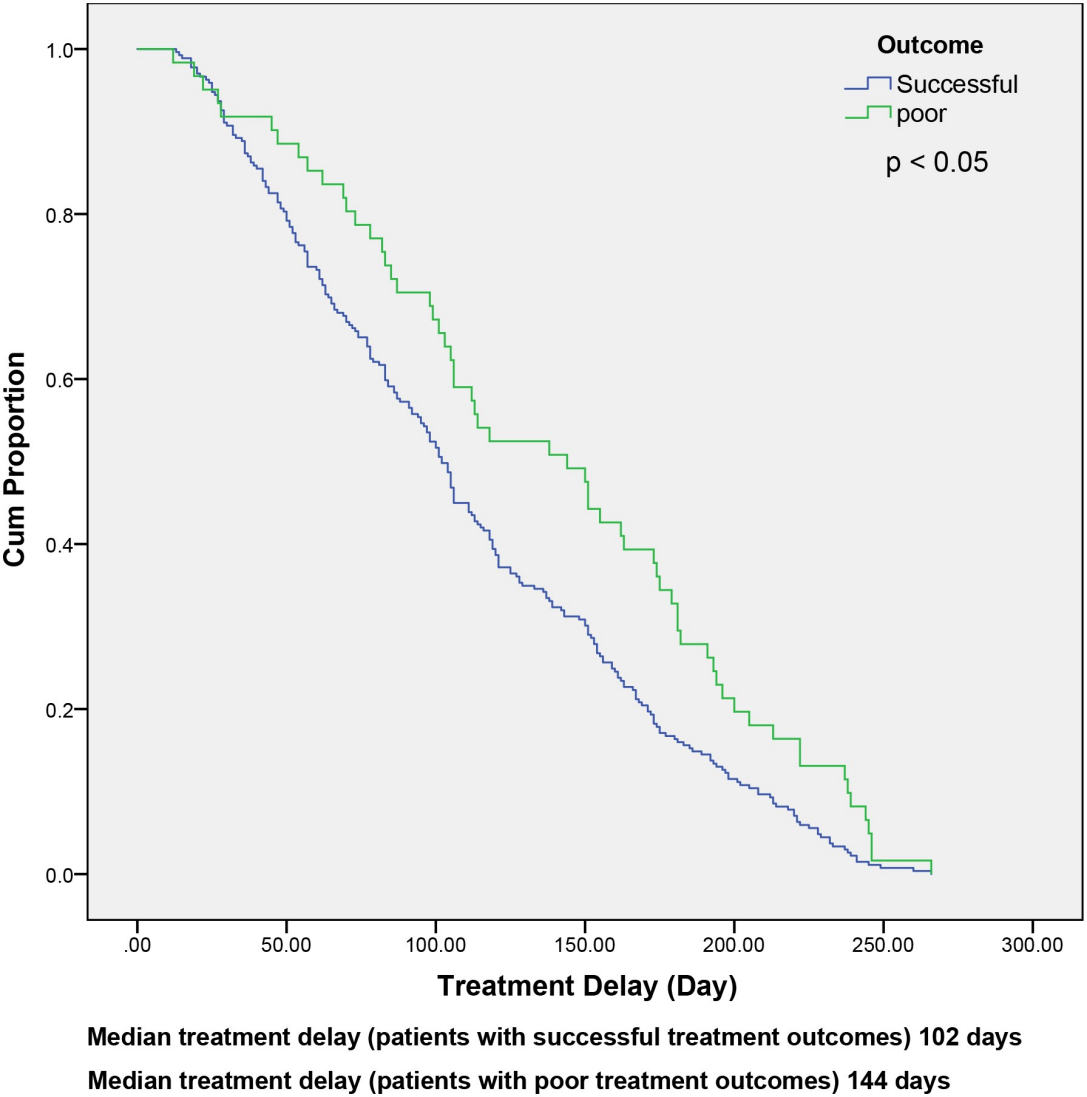
Treatment delays among patients with MDR-TB who had successful and poor outcomes.

As shown in Fig 3, the median treatment delay for MDR-TB patients with poor treatment outcomes was 144 days, which was longer than patients who achieved successful treatment outcomes (102 days). This difference was statistically significant (*p* < 0.05).

### Treatment adherence between long and short treatment delay groups

In comparing treatment adherence, the patients with long treatment delay appeared to have more incomplete monthly follow-up visits than those with short treatment delay (Table 5); however, the association between treatment adherence and delay in treatment initiation was not statistically significant (*p*-value = 0.05). Among patients with incomplete follow-up visits, it appears more cases with long treatment delay died during ≤ 4 monthly follow-up visits and 5–8 monthly follow-up visits. However, there were more incomplete follow-up-visit cases among the short delay treatment cases than those with long treatment delay after 9–12 monthly follow-up visits.

**Table 5:** Association between treatment adherence and treatment delay among patients with MDR-TB.

Treatment adherence		Long treatment delay	Short treatment delay	*p*-value
		n (%)	n (%)	
Complete follow up visits		134 (78.8)	139 (86.9)	0.05*
Incomplete follow up visits		36 (21.2)	21 (13.1)	
**Among incomplete follow up visits (n = 57) -**		(N = 36)	(N = 21)	
≤ 4 visits	Lost to follow-up	3 (8.3)	0 (0.0)	
	Died	13 (36.1)	3 (14.3)	
	Total	16 (44.4)	3 (14.3)	
5–8 visits	Lost to follow-up	2 (5.6)	2 (9.5)	
	Died	14 (38.9)	3 (14.3)	
	Total	16 (44.4)	5 (23.8)	
9–12 visits	Lost to follow-up	0 (0.0)	2 (9.5)	
	Died	3 (8.3)	7 (33.3)	
	Total	3 (8.3)	9 (42.9)	
13–16 visits	Lost to follow-up	0 (0.0)	0 (0.0)	
	Died	1 (2.8)	4 (19.0)	
	Total	1(2.8)	4 (19.0)	

* Chi-square Test

## Discussion

In this cohort study of MDR-TB patients, we assessed delays in treatment initiation and their effect on treatment outcomes. Regarding the period of delay in treatment initiation after MDR-TB diagnosis, the study findings indicated that the median post-diagnosis treatment delay was conspicuously long, at 105 days. Median delay times vary among countries that use the same definition of delay time, from 10 days in Bangladesh [22] to 27 days in South Africa [23]. After installing Xpert MTB/RIF facilities in the Yangon Region in 2012, unexpectedly higher numbers of RR-TB cases were identified in initial Xpert MTB/RIF screening. However, there were inadequate treatment initiation centers and limited healthcare staff to provide effective care. This was a problem of the capacity of the health system to deliver treatment, resulting in long waiting times for the initiation of MDR-TB treatment [15, 24].

Regarding the determinants of treatment delay, this study identified important patient-healthcare system interaction factors. The decision to start treatment being made by the Regional Expert DR-TB Committee led to a long waiting list and delays in treatment initiation. Consequently, patients might have been lost to follow-up between diagnosis and treatment initiation times. This situation could be avoided by expansion of decentralized MDR-TB treatment initiation centers, possibly with the availability of Xpert MTB/RIF diagnostic capacity. Moreover, the healthcare staff should be adequate for these expanded treatment initiation centers, to provide effective MDR-TB management within a short period post-diagnosis. It should be noted that, in 2015, treatment initiation, management of major adverse events, and treatment monitoring, were still centralized at the Regional TB Centre, although all 4 districts and 44 townships within the Yangon Region were covered by MDR-TB projects [13].

Patient factors, such as female sex, age > 30 years, and prior contact with patients who had MDR-TB, also influenced delays in treatment initiation. Family socioeconomic problems, stigma and discrimination, and cultural beliefs, make it more difficult for most female patients to access MDR-TB treatment. Women might also be concerned about the daily needs of their children and elderly family members. Hence, focusing comprehensive health education according to sex, and emphasizing healthcare-seeking behaviors, treatment adherence counseling, and motivation, should be provided during the pre-treatment period. In this respect, our finding differed from a study conducted in China, which suggested sex was not significantly associated with treatment delay [25].

Patients aged 31–50 years are generally considered to comprise the working-age population [14, 26]. These individuals are usually the main resources or income earners for their families. Workers might be concerned about the effects on their income due to absences from work and financial difficulties due to treatment, and they might have difficulty attending the Regional TB Centre to initiate TB treatment. Fear of financial hardship or catastrophic costs (e.g., for transport) and income loss during the treatment course might result in deferred treatment. In addition, most elderly patients are dependent and generally rely on their family members for their daily activities, which could result in delayed treatment among elderly patients. These individuals might also be concerned about the high pill burden, long treatment course, and adverse events, which are more common in elderly patients.

Prior contact with MDR-TB patients makes uninfected individuals more susceptible to contracting TB infection, and puts them at higher risk of developing MDR-TB. This situation may be controlled by accessing treatment soon after a confirmed diagnosis [27]. However, concerns about drug side-effects, long treatment duration, financial difficulties, and social discrimination might represent barriers to patients with a contact history from seeking prompt treatment. Other studies have revealed that financial burden, previously treated patients, history of chronic disease, clinical testing prior to treatment initiation, and long wait time for treatment, were related to delayed treatment initiation [22, 25, 28]. The early identification of contacts**,** initial home visits, contact tracing, and active TB screening of family members and contact persons, should be improved. Regular systematic clinical observation by monthly sputum smear and culture testing could also help detect individuals with new developing cases [27].

In exploring the effects of treatment delay on disease infectivity, severity, and treatment efficacy, our study confirmed these associations. In contrast with a study in Pakistan, which reported no association between treatment delay and culture conversion time [29], we found that delayed treatment initiation after diagnosis affected disease infectivity, severity, and treatment efficacy, and was a risk factor for high sputum smear grade, resistance to more than only isoniazid and rifampicin, and long sputum-culture conversion time. Sputum-smear microscopy is the most widely used method in most primary health care laboratories for assessing disease infectivity [30, 31]. High sputum smear grade is associated with a high bacterial load and chronicity, through extensive transmission in the environment, and is more prevalent among contacts of heavily smear-positive patients [21, 30]. Bacteriological smear and AFB culture examinations during the treatment period also serve as interim monitoring tools to assess treatment response. Sputum-culture conversion status is the most important indicator for the detection of live bacilli, and is used as a clinical tool to predict therapeutic efficacy and as an interim indicator of final treatment outcome [32].

An unanticipated finding from our study was the effect of treatment delay on treatment outcome. Interestingly, our study findings suggest that delayed treatment initiation is associated with poor treatment outcome, albeit not significantly after adjustment for other risk factors. This non-significant association is consistent with a previous study conducted in Switzerland [33]. Theoretically, the longer the delay in initiating treatment, the poorer the treatment outcome; however, further investigation of this issue is needed. It should be noted that treatment outcomes were partially related to treatment adherence. In this study, more patients with long treatment delays were lost or died during the 8 monthly follow-up visits, compared with those with short treatment delay. There seem to be more lost to follow-up or dead cases among short treatment delays at later monthly visits. Regarding other factors associated with poor treatment outcome, we found that patients who were underweight at the time of initiating treatment had a higher risk of severe clinical presentation and poor outcome [34]; this finding is consistent with those of other studies [35–37]. In the present study, most patients were workers, and some had poor nutrition due to financial constraints. Loss of appetite, nausea, and vomiting aggravated by second-line drugs, and the disease itself contribute to weight loss and nutritional deficiency. This can be addressed by the use of ancillary drugs to alleviate gastrointestinal side effects and by providing nutritional supplementation as an additional support [38].

Patients with HIV coinfection were also likely to have poor outcomes, which is consistent with the findings of studies conducted in other countries [35, 39]. In Myanmar, HIV testing among TB patients has been scaled up rapidly, but slower progress has been made in TB screening of people living with HIV. Referral and feedback systems are additional challenges in TB/HIV collaborative activities, and the poor accessibility of antiretroviral therapy (ART) remains a limitation among patients with HIV coinfection [13]. Patients with MDR-TB/HIV coinfection should be provided with early diagnosis, timely access to second-line anti-TB drugs, motivational counseling, and early ART, as soon as MDR-TB treatment is tolerated, regardless of CD4 count [13, 40]. Close monitoring of treatment side effects, clinical management and prophylaxis of opportunistic infections, nutritional support, and additional socioeconomic support are also important for treatment adherence [11]. The findings of our study also indicated that patients with high sputum smear grading were more likely to have poor treatment outcomes. An advanced disease condition with a higher grade of sputum positivity at the initiation of treatment could result in disease transmission to the community [41]. However, a study conducted in Latvia reported that high smear grade was not identified as a predictor of poor treatment outcome [36].

Myanmar is currently moving toward a more streamlined diagnostic network through intensified approaches focusing on improved access to quality diagnostic services, with adequate facilities and acceleration of prompt treatment. By 2020, the NTP expects that all patients with MDR-TB in the country can be enrolled in treatment within 2 weeks of diagnosis [11]. Xpert MTB/RIF machines are currently available in 69 laboratories throughout the Region/State and district levels, for use as the primary diagnostic tool for RR/MDR-TB. Screened RR-TB patients are treated with a standard regimen, according to the guidelines for DR-TB management, updated in 2017 [24]. DST methods are only used in strongly suspected cases or those with discordant Xpert MTB/RIF results [11]. Moreover, with financial support, primarily from the Global Fund and 3MDG, the government of Myanmar is directing much of its expenditure towards the implementation of second-line drugs and infrastructure. It is anticipated that such investments could eliminate the waiting time for decisions of the Expert DR-TB Committee, so that all patients with confirmed MDR/RR-TB who are registered in the NTP can promptly begin a standard regimen.

The Ministry of Health and Sports is also adopting universal health care, to extend free coverage for services to all patients and thereby reduce the catastrophic costs for these patients. Initiated in 2015, a standard compensation package of 30 USD per month is provided to patients to cover travel allowance and nutritional support, so as to achieve good treatment adherence [11, 15]. Current approaches also include expansion of decentralized MDR-TB treatment initiation centers with quality diagnostic facilities, management of minor drug side-effects, as well as manpower recruitment for expanded treatment centers.

### Limitations

The current study was based on secondary data collected from the databases of Yangon Regional TB Centre and the data were extracted manually from patient treatment cards by the researchers. Due to the limited structure of the data-recording system in the national databases in 2014, some sociodemographic predictors, such as occupation, education, alcohol, smoking, distance and travelling time to treatment center, as well as serious adverse events, were not included in the treatment cards. Thus, it is possible that we may have omitted several important factors associated with treatment delay and treatment outcomes. Moreover, this study focused on adult patients aged > 18 years who were registered at Yangon Regional TB Center; therefore, the results among other age groups and in other Myanmar settings during the study period might differ.

## Conclusions

We found that a large proportion of patients with MDR-TB had a long delay in post-diagnosis treatment initiation. We also found that long treatment delay affects disease infectivity, severity, and treatment efficacy. The study results showed that inadequate MDR-TB treatment initiation centers, centralization of treatment initiation, limitations in human resources, were health-system factors affecting timely treatment initiation and implementation of an effective TB control program. There is a need for immediate interventions that may reduce treatment delay and improve treatment outcomes, including scaling up diagnostic capacity with Xpert MTB/RIF in all townships, and expansion of decentralized MDR-TB treatment-initiation centers. Other important measures involve ensuring a productive health workforce with trained health personnel, together with providing reimbursement to BHS for conducting patient tracking, systematic contact tracing, and active TB screening. Additionally, at patient and community levels, providing health education and treatment adherence counseling to raise public awareness of early treatment, as well as continuation of a standard support package to all patients, will help yield good treatment adherence. Myanmar is currently moving towards implementing many interventions to minimize treatment delay for patients with MDR-TB; it is yet to elucidate the anticipated impacts.

## Supporting information

S1 File MDR-TB treatment card. (DOCX)
