# Supplementary material for: Delay in treatment initiation and treatment outcomes among adult patients with multidrug-resistant tuberculosis at Yangon Regional Tuberculosis Centre, Myanmar: A retrospective study
Source: PLoS One. 2018 Dec 31;13(12):e0209932. doi: 10.1371/journal.pone.0209932 (PMC6312206; doi:10.1371/journal.pone.0209932)
Supplement: S1 File — (DOCX) [file pone.0209932.s001.docx]

**NATIONAL TUBERCULOSIS PROGRAMME**

**MDR-TB Treatment Card**

Name: _________________________________________________________

Sex: [ ] M [ ] F

Age: ____________ Date of birth: ______/_______/______________

Initial weight (kg): ______________ Height (cm): _______________

Site: [ ] Pulmonary [ ] Extra-pulmonary [ ] Both

If extra-pulmonary, specific site: ____________________________________

MDR TB registration number: ______________________________________

Date of registration: ______/______/______________

Township TB number:

Date of township TB registration: ____/____/_________________

Address: _______________________________________________________

Township/District: _______________________________________________

Treatment/centre: ________________________________________________

Name of DOT Provider: ___________________________________________

DOT Supervisor: _________________________________________________

| **No** | **Registration group** | **Select**  **one only** |
| --- | --- | --- |
| 1 | New |  |
| 2 | Relapse |  |
| 3 | Treatment after default |  |
| 4 | Treatment after failure of Category I treatment |  |
| 5 | Treatment after failure of Category II treatment |  |
| 6 | Treatment after failure with the Standard MDR-TB regimen |  |
| 7 | Other |  |

*Backlog of cases who have waited with no or inadequate treatment for a period of time

Contact of MDR-TB case: [ ] Yes [ ] No

| **HIV information** |
| --- |
| HIV testing done: [ ] Y [ ] N [ ] unknown |
| Date of test ____/____/________ Results: |
| Started on ART: [ ] Y [ ] N Date ____/____/_______ |
| Started on CPT: [ ] Y [ ] N Date ____/____/_______ |

ART= antiretroviral therapy; CPT = co-trimoxazole preventive therapy

**Previous tuberculosis treatment episodes**

| **Previous**  **Township TB**  **No./township** | **Start date**  **(if unknown,**  **put year)** | **Regimen**  **(in drug**  **abbreviations)** | **Outcome** |
| --- | --- | --- | --- |
|  |  |  |  |
|  |  |  |  |
|  |  |  |  |
|  |  |  |  |
|  |  |  |  |

Used second-line drugs previously? [ ] Yes [ ] No

If yes, specify: _____________________________________

**Drug abbreviations**

**First-line drugs Second-line drugs**

H= Isoniazid Am= Amikacin

R= Rifampicin Km= Kanamycin

E= Ethambutol Cm= Capreomycin

Z= Pyrazinamide Cfx= Ciprofloxacin

S= Streptomycin Ofx= Ofloxacin

(Th= Thioacetazone) Lfx= Levofloxacin

Mfx= Moxifloxacin

Gfx= Gatifloxacin

Pto= Prothionamide

Eto= Ethionamide

Cs= Cycloserine

PAS= P-aminosalicylic Na

**District/Township MDR-TB Committee recommendation**

| **Date** | **Decision** | **Next date** |
| --- | --- | --- |
|  |  |  |
|  |  |  |
|  |  |  |


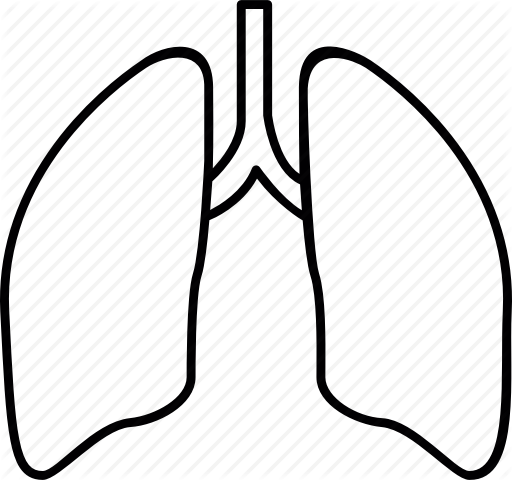


Date of Xray……………………..


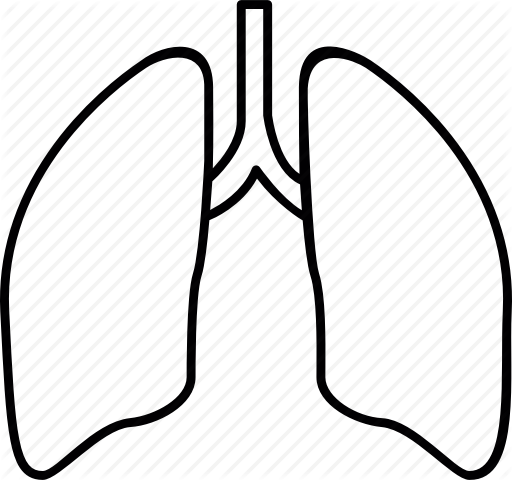


Date of Xray……………………..


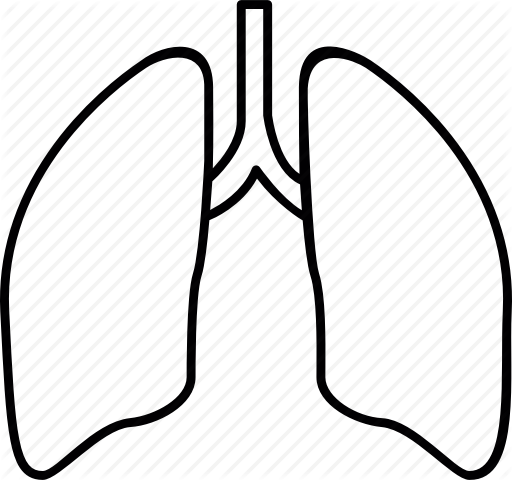


Date of Xray……………………..


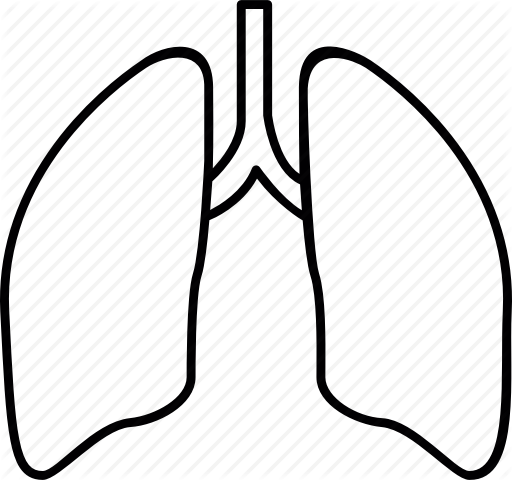


Date of Xray……………………..

| **Month**  **No.** | **Sputum Smear Microscopy** | | | **Culture** | | | **Urea** | **Serum**  **Creatinine** | **LFT** | **CP** | **Serum**  **Uric**  **Acid** | **TSH** | **ECG** | **Electrolytes** | **Blood**  **sugar** |
| --- | --- | --- | --- | --- | --- | --- | --- | --- | --- | --- | --- | --- | --- | --- | --- |
|  | **Date** | **Sample**  **No.** | **Grading** | **Date** | **Sample**  **No.** | **Grading** |  |  |  |  |  |  |  |  |  |
|  |  |  |  |  |  |  |  |  |  |  |  |  |  |  |  |
|  |  |  |  |  |  |  |  |  |  |  |  |  |  |  |  |
|  |  |  |  |  |  |  |  |  |  |  |  |  |  |  |  |
|  |  |  |  |  |  |  |  |  |  |  |  |  |  |  |  |
|  |  |  |  |  |  |  |  |  |  |  |  |  |  |  |  |
|  |  |  |  |  |  |  |  |  |  |  |  |  |  |  |  |
|  |  |  |  |  |  |  |  |  |  |  |  |  |  |  |  |
|  |  |  |  |  |  |  |  |  |  |  |  |  |  |  |  |
|  |  |  |  |  |  |  |  |  |  |  |  |  |  |  |  |
|  |  |  |  |  |  |  |  |  |  |  |  |  |  |  |  |
|  |  |  |  |  |  |  |  |  |  |  |  |  |  |  |  |
|  |  |  |  |  |  |  |  |  |  |  |  |  |  |  |  |
|  |  |  |  |  |  |  |  |  |  |  |  |  |  |  |  |
|  |  |  |  |  |  |  |  |  |  |  |  |  |  |  |  |

**Drug susceptibility testing (DST) results**

| **Date** | S | H | R | E | **Pto/Eto** | **Km/Amk** | **Cm** | **Fq** | **Other** | **Other** | **Other** |
| --- | --- | --- | --- | --- | --- | --- | --- | --- | --- | --- | --- |
|  |  |  |  |  |  |  |  |  |  |  |  |
|  |  |  |  |  |  |  |  |  |  |  |  |

R = resistant, S= susceptible, C = contaminated

**MDR-TB REGIMEN** (date treatment started and dosage (mg), frequency of dose, change of dosage, and cessation of drugs):

| **Date** | **H** | **R** | **Z** | **E** | **S** | **Km** | **Am** | **Cm** | **FQ** | **Pto/Eto** | **Cs** | **PAS** | **Other** | **Comments** |
| --- | --- | --- | --- | --- | --- | --- | --- | --- | --- | --- | --- | --- | --- | --- |
|  |  |  |  |  |  |  |  |  |  |  |  |  |  |  |
|  |  |  |  |  |  |  |  |  |  |  |  |  |  |  |
|  |  |  |  |  |  |  |  |  |  |  |  |  |  |  |
|  |  |  |  |  |  |  |  |  |  |  |  |  |  |  |
|  |  |  |  |  |  |  |  |  |  |  |  |  |  |  |
|  |  |  |  |  |  |  |  |  |  |  |  |  |  |  |
|  |  |  |  |  |  |  |  |  |  |  |  |  |  |  |
|  |  |  |  |  |  |  |  |  |  |  |  |  |  |  |
|  |  |  |  |  |  |  |  |  |  |  |  |  |  |  |

(od = Once a day, bd = 12 hourly: morning and evening doses)

**ADMINISTRATION OF DRUGS (one line per month):**

| **Month** | **1** | **2** | **3** | **4** | **5** | **6** | **7** | **8** | **9** | **10** | **11** | **12** | **13** | **14** | **15** | **16** | **17** | **18** | **19** | **20** | **21** | **22** | **23** | **24** | **25** | **26** | **27** | **28** | **29** | **30** | **31** | **Weight (kg)** |
| --- | --- | --- | --- | --- | --- | --- | --- | --- | --- | --- | --- | --- | --- | --- | --- | --- | --- | --- | --- | --- | --- | --- | --- | --- | --- | --- | --- | --- | --- | --- | --- | --- |
|  |  |  |  |  |  |  |  |  |  |  |  |  |  |  |  |  |  |  |  |  |  |  |  |  |  |  |  |  |  |  |  |  |
|  |  |  |  |  |  |  |  |  |  |  |  |  |  |  |  |  |  |  |  |  |  |  |  |  |  |  |  |  |  |  |  |  |
|  |  |  |  |  |  |  |  |  |  |  |  |  |  |  |  |  |  |  |  |  |  |  |  |  |  |  |  |  |  |  |  |  |
|  |  |  |  |  |  |  |  |  |  |  |  |  |  |  |  |  |  |  |  |  |  |  |  |  |  |  |  |  |  |  |  |  |
|  |  |  |  |  |  |  |  |  |  |  |  |  |  |  |  |  |  |  |  |  |  |  |  |  |  |  |  |  |  |  |  |  |
|  |  |  |  |  |  |  |  |  |  |  |  |  |  |  |  |  |  |  |  |  |  |  |  |  |  |  |  |  |  |  |  |  |
|  |  |  |  |  |  |  |  |  |  |  |  |  |  |  |  |  |  |  |  |  |  |  |  |  |  |  |  |  |  |  |  |  |
|  |  |  |  |  |  |  |  |  |  |  |  |  |  |  |  |  |  |  |  |  |  |  |  |  |  |  |  |  |  |  |  |  |
|  |  |  |  |  |  |  |  |  |  |  |  |  |  |  |  |  |  |  |  |  |  |  |  |  |  |  |  |  |  |  |  |  |

Mark in the boxes: √ = directly observed

x = not supervised

0 = drugs not taken

**ADMINISTRATION OF DRUGS (one line per month):**

| **Month** | **1** | **2** | **3** | **4** | **5** | **6** | **7** | **8** | **9** | **10** | **11** | **12** | **13** | **14** | **15** | **16** | **17** | **18** | **19** | **20** | **21** | **22** | **23** | **24** | **25** | **26** | **27** | **28** | **29** | **30** | **31** | **Weight (kg)** |
| --- | --- | --- | --- | --- | --- | --- | --- | --- | --- | --- | --- | --- | --- | --- | --- | --- | --- | --- | --- | --- | --- | --- | --- | --- | --- | --- | --- | --- | --- | --- | --- | --- |
|  |  |  |  |  |  |  |  |  |  |  |  |  |  |  |  |  |  |  |  |  |  |  |  |  |  |  |  |  |  |  |  |  |
|  |  |  |  |  |  |  |  |  |  |  |  |  |  |  |  |  |  |  |  |  |  |  |  |  |  |  |  |  |  |  |  |  |
|  |  |  |  |  |  |  |  |  |  |  |  |  |  |  |  |  |  |  |  |  |  |  |  |  |  |  |  |  |  |  |  |  |
|  |  |  |  |  |  |  |  |  |  |  |  |  |  |  |  |  |  |  |  |  |  |  |  |  |  |  |  |  |  |  |  |  |
|  |  |  |  |  |  |  |  |  |  |  |  |  |  |  |  |  |  |  |  |  |  |  |  |  |  |  |  |  |  |  |  |  |
|  |  |  |  |  |  |  |  |  |  |  |  |  |  |  |  |  |  |  |  |  |  |  |  |  |  |  |  |  |  |  |  |  |
|  |  |  |  |  |  |  |  |  |  |  |  |  |  |  |  |  |  |  |  |  |  |  |  |  |  |  |  |  |  |  |  |  |
|  |  |  |  |  |  |  |  |  |  |  |  |  |  |  |  |  |  |  |  |  |  |  |  |  |  |  |  |  |  |  |  |  |
|  |  |  |  |  |  |  |  |  |  |  |  |  |  |  |  |  |  |  |  |  |  |  |  |  |  |  |  |  |  |  |  |  |
|  |  |  |  |  |  |  |  |  |  |  |  |  |  |  |  |  |  |  |  |  |  |  |  |  |  |  |  |  |  |  |  |  |
|  |  |  |  |  |  |  |  |  |  |  |  |  |  |  |  |  |  |  |  |  |  |  |  |  |  |  |  |  |  |  |  |  |
|  |  |  |  |  |  |  |  |  |  |  |  |  |  |  |  |  |  |  |  |  |  |  |  |  |  |  |  |  |  |  |  |  |
|  |  |  |  |  |  |  |  |  |  |  |  |  |  |  |  |  |  |  |  |  |  |  |  |  |  |  |  |  |  |  |  |  |
|  |  |  |  |  |  |  |  |  |  |  |  |  |  |  |  |  |  |  |  |  |  |  |  |  |  |  |  |  |  |  |  |  |
|  |  |  |  |  |  |  |  |  |  |  |  |  |  |  |  |  |  |  |  |  |  |  |  |  |  |  |  |  |  |  |  |  |

Mark in the boxes: √ = directly observed

x = not supervised

0 = drugs not taken

Comments __________________________________________________________________________

___________________________________________________________________________________

___________________________________________________________________________________

___________________________________________________________________________________

___________________________________________________________________________________

| **Outcome** | **Mark one** | **Date** |
| --- | --- | --- |
| Cured |  |  |
| Completed |  |  |
| Failed |  |  |
| Died |  |  |
| Lost to follow-up |  |  |
| Not evaluated |  |  |
